# *Piper sarmentosum *inhibits ICAM-1 and Nox4 gene expression in oxidative stress-induced human umbilical vein endothelial cells

**DOI:** 10.1186/1472-6882-11-31

**Published:** 2011-04-16

**Authors:** Azizah Ugusman, Zaiton Zakaria, Chua Kien Hui, Nor Anita Megat Mohd Nordin

**Affiliations:** 1 Department of Physiology, Faculty of Medicine, Universiti Kebangsaan Malaysia Medical Centre, Jalan Raja Muda Abdul Aziz, 50300 Kuala Lumpur, Malaysia

## Abstract

**Background:**

Aqueous extract of *Piper sarmentosum *(AEPS) is known to possess antioxidant and anti-atherosclerotic activities but the mechanism responsible for it remains unclear. In early part of atherosclerosis, nuclear factor-kappa B (NF-κB) induces the expression of cellular adhesion molecules such as vascular cell adhesion molecule-1 (VCAM-1), intracellular adhesion molecule-1 (ICAM-1) and E-selectin. NADPH oxidase 4 (Nox4) is the predominant source of superoxide in the endothelial cells whereas superoxide dismutase 1 (SOD1), catalase (CAT) and glutathione peroxidase (GPx) are the antioxidant enzymes responsible for inactivating reactive oxygen species. The present study aimed to investigate the effects of AEPS on the gene expression of NF-κB, VCAM-1, ICAM-1, E-selectin, Nox4, SOD1, CAT and GPx in cultured human umbilical vein endothelial cells (HUVECs).

**Methods:**

HUVECs were divided into four groups:- control; treatment with 180 μM hydrogen peroxide (H_2_O_2_); treatment with 150 μg/mL AEPS and concomitant treatment with AEPS and H_2_O_2 _for 24 hours. Total RNA was extracted from all the groups of HUVEC using TRI reagent. Subsequently, qPCR was carried out to determine the mRNA expression of NF-κB, VCAM-1, ICAM-1, E-selectin, Nox4, SOD1, CAT and GPx. The specificity of the reactions was verified using melting curve analysis and agarose gel electrophoresis.

**Results:**

When stimulated with H_2_O_2_, HUVECs expressed higher level of ICAM-1 (1.3-fold) and Nox4 (1.2-fold) mRNA expression. However, AEPS treatment led to a reduction in the mRNA expression of ICAM-1 (p < 0.01) and Nox4 (p < 0.05) in the H_2_O_2_-induced HUVECs. AEPS also upregulated the mRNA expression of SOD1 (p < 0.05), CAT (p < 0.01) and GPx (p < 0.05) in oxidative stress-induced HUVECs. There was no significant change in the mRNA expression of VCAM-1 and E-selectin.

**Conclusion:**

The expressional suppression of ICAM-1 and Nox4 and induction of antioxidant enzymes might be an important component of the vascular protective effect of AEPS.

## Background

Atherosclerosis has been recognized as a chronic inflammatory disease and oxidative stress plays a pivotal role in its initiation and progression [1]. Endothelial dysfunction is considered to be an early marker for atherosclerosis [2]. Evidence suggests that increased production of reactive oxygen species (ROS) and vascular inflammation play important roles in endothelial dysfunction.

Vascular disorders, through over expression of adhesion molecules and cytokines are involved in the development of atherosclerosis. Endothelial cells in human atherosclerotic lesions have increased cell adhesion molecules expression such as intercellular adhesion molecule-1 (ICAM-1), vascular cell adhesion molecule-1 (VCAM-1) and endothelial selectin (E-selectin) [3,4]. The adhesion of monocytes to the arterial wall and their subsequent infiltration and differentiation into macrophages are the key events in the development of atherosclerosis. Nuclear factor-kappa B (NF-κB) is known to play a critical role in the development of inflammatory response by upregulating the expression of VCAM-1, ICAM-1 and E-selectin [5]. It has been suggested that NF-κB is an oxidative stress-responsive transcription factor. Antioxidants and free radical scavengers such as vitamin E derivatives, N-acetyl-cysteine and thiol reagents inhibit the activation of NF-κB, strongly supporting the idea that reactive oxygen species (ROS) are involved in the activation process [6].

Under oxidative stress, macrophages generate ROS such as superoxides, leading to LDL oxidation [7]. In the vascular wall, ROS can be produced by several enzyme systems including NADPH oxidases, xanthine oxidase, uncoupled endothelial nitric oxide synthase, lipoxygenases and myeloperoxidase [8]. Although all these enzymes can contribute to oxidative stress, NADPH oxidases (Nox) are the predominant source of ROS in the vasculature. In diseased human coronary arteries, about 60% of total vascular superoxide is derived from Nox [9]. Vascular tissues express the Nox isoforms Nox1, Nox2, Nox4 and Nox5. In human umbilical vein endothelial cells (HUVECs), the expression level of Nox4 is 100-fold higher than that of Nox1, Nox2 or Nox5, suggesting that Nox4 is the major source of ROS in these cells [10]. Increased Nox4 expression is associated with early progression of atherosclerotic plaque [11].

To protect cells from the damage caused by ROS, organisms have evolved several defense mechanisms to rapidly and efficiently remove ROS. This includes the antioxidant enzymes such as superoxide dismutase (SOD), catalase (CAT) and glutathione peroxidase (GPx). SOD catalyzes the dismutation of superoxide to hydrogen peroxide (H_2_O_2_) while CAT and GPx convert H_2_O_2 _to water [12].

**Figure 1:**
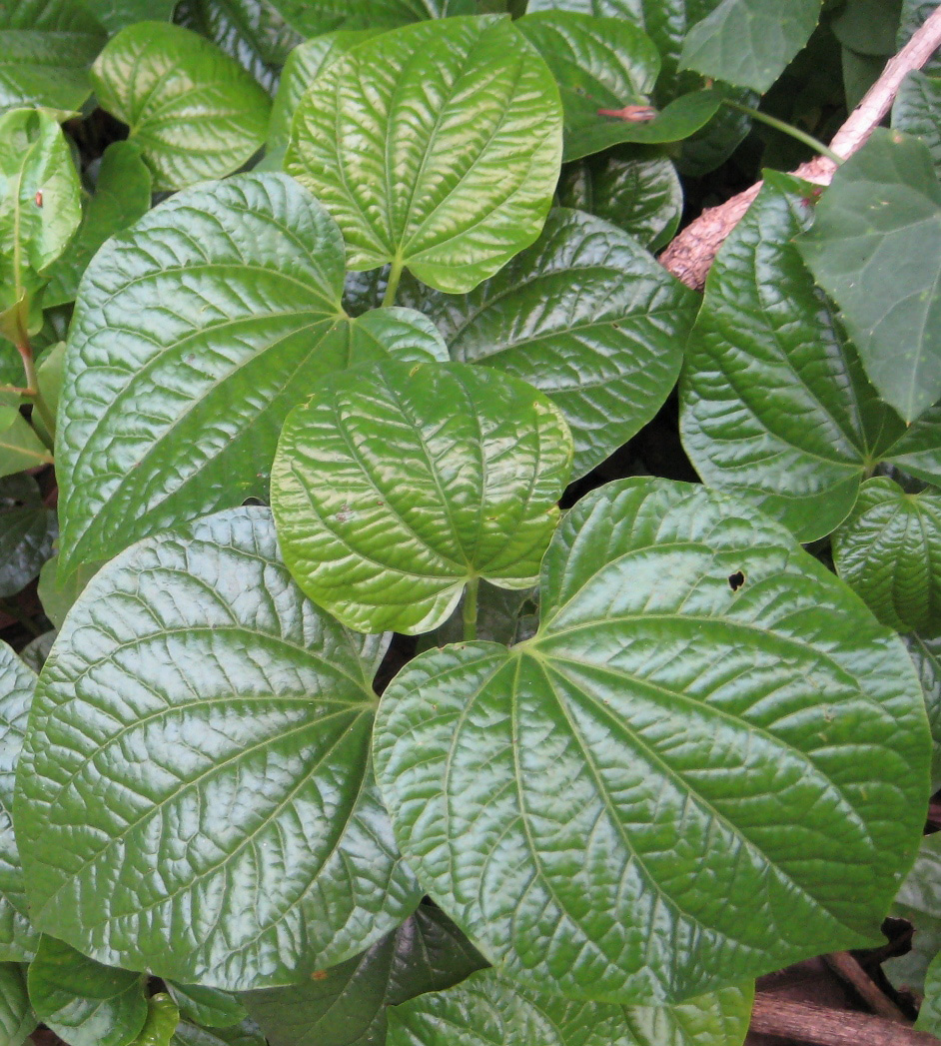
***Piper sarmentosum *leaves**.

*Piper sarmentosum *(Figure 1) is a herbaceous plant that is commonly found in the tropical regions such as the Southeast Asia. The plant extracts have been reported to possess pharmacological properties like anti-tuberculous [13], anti cancer [14], hypoglycaemic [15], anti-malarial [16], anti-nociceptive and anti-inflammatory [17]. As per recent research reports, the aqueous extracts of *Piper sarmentosum *(AEPS) leaves have been reported to improve endothelial function by promoting nitric oxide production in HUVECs [18]. Various extracts prepared from *Piper sarmentosum *leaves have exhibited potent effects as antioxidant in modulating oxidative stress in H_2_O_2_-induced HUVECs [19]. Moreover, administration of AEPS reduced atherosclerotic lesion in hypercholesterolemic rabbits [20]. Based on the cardiovascular protective effects of AEPS mentioned above, the present study was designed to investigate the effects of AEPS on the gene expression of NF-κB, cellular adhesion molecules (VCAM-1, ICAM-1 and E-selectin), Nox4 and antioxidant enzymes (SOD1, CAT, GPx) in HUVECs.

## Methods

### Preparation of aqueous extract of Piper sarmentosum

Leaves of *Piper sarmentosum *were collected in Sungai Buloh, Malaysia and identified by a plant taxonomist from Forest Research Institute of Malaysia (voucher specimen: FRI 45870). The leaves were washed with tap water, cut into small pieces, sun-dried and grounded to powder form. Ten percent of AEPS was prepared by soaking 100 g of the powdered leaves in 900 ml of purified water and incubated in a high speed mixer at 80°C for 3 hours. After being left to cool, the extract was filtered using a mesh and further concentrated. The aqueous extract was then freeze-dried to powdered form of the extract and kept at 4°C until the experiments.

### Cell culture and treatment protocols

Human umbilical cords were obtained under sterile condition. Informed consent was obtained from each subject and the present study was approved by the Ethical Research Committee of Universiti Kebangsaan Malaysia Medical Center (Project Code: FF-148-2009). HUVECs were obtained from umbilical cord veins by 0.1% Collagenase Type I (Gibco-Invitrogen Corp., Grand Island, N.Y.) digestion. Cells were grown in medium 200 (Cascade Biologics, USA) supplemented with LSGS (low serum growth supplement; Cascade Biologics, USA) at 37°C in a humidified atmosphere of 5% CO_2 _and 95% air. HUVECs were confirmed by the typical endothelial cell cobblestone morphology and the positive expressions of vonWillebrand factor and CD31 in immunocytochemistry. The culture medium was changed every other day until the cells reached confluence. HUVECs from passage 3 at 80% confluency were used for this experiment. The cells were divided into four groups:- control; treatment with 180 μM H_2_O_2_; treatment with 150 μg/mL AEPS and concomitant treatment with 150 μg/mL AEPS and 180 μM H_2_O_2_. All treatments were given for 24 hours. The dose of H_2_O_2 _used was based on the IC_50 _of H_2_O_2 _while the dose of AEPS was based on the EC_50 _of AEPS adopted from a previous study [19]. The rationale to use H_2_O_2 _in this study was to induce oxidative stress. Oxidative stress has been associated with endothelial dysfunction which later progresses into the development of atherosclerosis.

### Quantitative reverse transcription polymerase chain reaction (qPCR)

After treatment for 24 hours, total RNA from HUVECs was extracted using TRI Reagent (Molecular Research Center, Cincinnati, USA) as previous research protocol [21]. Polyacryl carrier (Molecular Research Center, Cincinnati, USA) was added to precipitate the total RNA. Extracted RNA pellet was then washed with 75% ethanol and dried prior to dissolving it in RNase and DNase free water (Invitrogen, Carlsbad, USA). Extracted total RNA was assessed for its purity and quantity using Nanodrop ND-100 spectrophotometer (Wilmington DE, USA) and stored at -80°C before use. Complimentary DNA (cDNA) was synthesized using SuperScript III First-Strand Synthesis SuperMix (Invitrogen, Carlsbad, USA). A total of 20 μl of volume reaction which consisted of 10 μl of 2X RT Reaction Mix, 2 μl of RT Enzyme, 5 μl of total RNA and 3 μl of DEPC-treated water was incubated at 25°C for 10 minutes for primer annealing then at 50°C for 30 minutes for reverse transcription. Following this, the reaction was terminated at 85°C for 5 minutes, chilled on ice for 1 minute and 1 μl of *E. coli *RNase H was added to the mixture. The cDNA was further incubated at 37°C for 20 minutes and stored at -20°C until use. Subsequently qPCR was carried out to determine the mRNA expression of NF-κB, VCAM-1, ICAM-1, E-selectin, Nox4, SOD1, CAT and GPx. Glycerylaldehyde-3-phosphate dehydrogenase (GAPDH) was used as the reference gene. Primer 3 software [22] was used to design the primers from NIH GenBank database. The primer sequences for NF-κB, VCAM-1, ICAM-1, E-selectin, Nox4, SOD1, CAT and GPx are as listed in Table 1. The qPCR reaction was performed with 1 μl of cDNA, 5 μM of each forward and reverse primer and 12.5 μl of IQ SYBR Green Supermix (Bio-Rad, USA) in BioRad iCycler (Bio-Rad, USA) with reaction profile of: 40 cycles of 95°C (10 seconds) and 61°C (30 seconds). The reaction kinetic of each primer set and protocol was verified with melting profile and product size was further confirmed with 2% agarose gel electrophoresis stained with ethidium bromide (Sigma, St Louis, USA). The threshold cycle (C_T_) value was determined and the relative mRNA expression of the genes was calculated as follows: 2∆∆^CT ^with ∆∆C_T _= C_T _GAPDH - C_T _gene of interest.

**Table 1:** List of primers for qPCR analysis

Target mRNA	Primer sequence	Genbank accession no	PCR product size (bp)
**GAPDH**	F:tccctgagctgaacgggaag R:ggaggagtgggtgtcgctgt	NM_002046	217
**NF-κB**	F: agtgcagaggaaacgtcagaa R: cattttaccacttggcaggaa	NM_003998	163
**VCAM-1**	F: agttgaaggatgcgggagtat R: ggatgcaaaatagagcacgag	NM_001078	143
**ICAM-1**	F: cagtcacctatggcaacgact R: ctctggcttcgtcagaatcac	NM_000201	179
**E-selectin**	F: gtttggtgaggtgtgctcatt R: cattttaccacttggcaggaa	NM_000450	163
**Nox4**	F: cagaaggttccaagcaggag R: gttgagggcattcaccagat	NM_016931	129
**SOD1**	F: tggccgatgtgtctattgaa R: cacctttgcccaagtcatct	NM_000454	108
**CAT**	F: gccattgccacaggaaagta R: ccttggtgagatcgaatgga	NM_001752	103
**GPx**	F: ccaagctcatcacctggtct R: tcgatgtcaatggtctggaa	NM_000581	198

### Statistical analysis

Data was tested for normality using Kolmogorov-Smirnov test and all variables were normally distributed. Data was expressed as means of fold change ± SEM. Statistical analysis between two groups was performed using paired t-test in SPSS version 16.0 software. Values of p < 0.05 were considered statistically significant.

## Results

### Effects of AEPS on NF-κB mRNA expression in HUVECs

**Figure 2:**
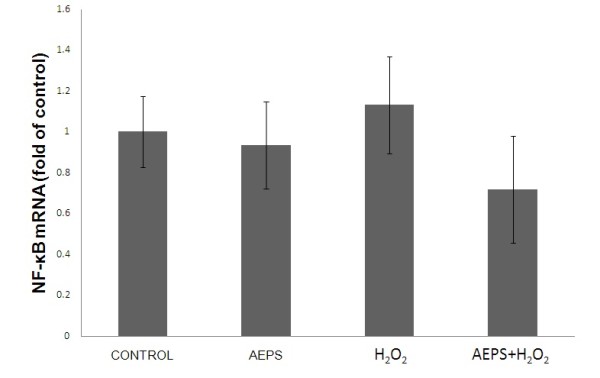
**NF-κB mRNA expression in HUVECs**. Figure 2 represents the bar chart showing NF-κB mRNA expression in control, AEPS, H_2_O_2 _and AEPS + H_2_O_2 _groups. Both AEPS and H_2_O_2 _did not cause significant changes in NF-κB mRNA expression in HUVEC. Values are means ± SEM of n = 6.

Both AEPS and H_2_O_2 _did not show any significant changes in the mRNA expression of NF-κB (Figure 2).

### Effects of AEPS on VCAM-1, ICAM-1 and E-selectin mRNA expression in HUVECs

**Figure 3:**
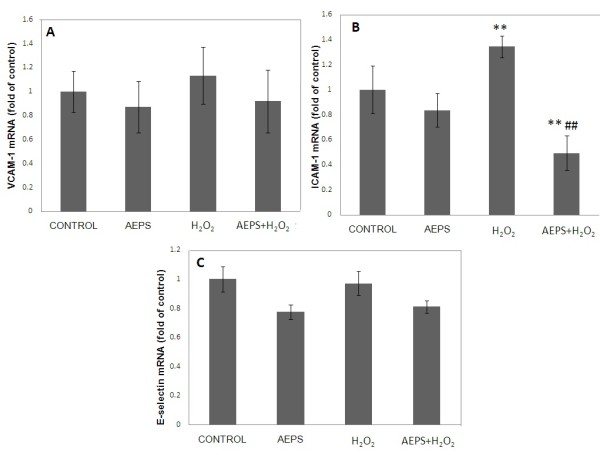
**VCAM-1, ICAM-1 and E-selectin mRNA expression in HUVECs**. Figure 3 represents the bar chart showing VCAM-1 (A), ICAM-1 (B) and E-selectin (C) mRNA expression in control, AEPS, H_2_O_2 _and AEPS + H_2_O_2 _groups. HUVECs treated with H_2_O_2 _showed a significantly higher level of ICAM-1 mRNA expression. The H_2_O_2_-induced ICAM-1 mRNA expression was significantly down regulated by AEPS. Data are denoted as mean ± SEM of n = 6. (**) p < 0.01 vs. control; (##) p < 0.01 vs. H_2_O_2_.

Treatment with AEPS alone did not show a significant change in ICAM-1 expression (Figure 3B). HUVECs treated with H_2_O_2 _showed a significantly higher (1.3-fold) level of ICAM-1 mRNA expression compared to the control group. Concomitant treatment of HUVECs with both AEPS and H_2_O_2 _resulted in a down regulation of ICAM-1 mRNA expression than the control and H_2_O_2 _groups. However, there was no significant change in the mRNA expressions of VCAM-1 and E-selectin in response to AEPS and H_2_O_2 _(Figure 3A, 3C).

### Effects of AEPS on Nox4 mRNA expression in HUVECs

**Figure 4:**
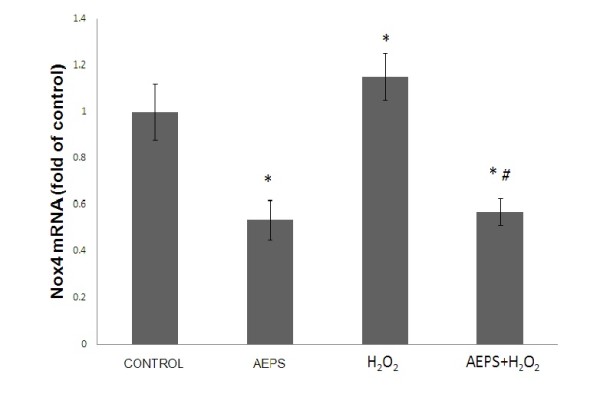
**Nox4 mRNA expression in HUVECs**. Figure 4 represents the bar chart showing Nox4 mRNA expression in control, AEPS, H_2_O_2 _and AEPS + H_2_O_2 _groups. HUVECs treated with AEPS had lower Nox4 mRNA expression while H_2_O_2 _caused a higher Nox4 mRNA expression. Data are denoted as mean ± SEM of n = 6. (*) p < 0.05 vs. control; (#) p < 0.05 vs. H_2_O_2_.

The aqueous extract of PS significantly reduced Nox4 mRNA expression in HUVECs compared to the control group (Figure 4). When stimulated with H_2_O_2_, HUVECs expressed higher (1.2-fold) level of Nox4 mRNA expression. However, the H_2_O_2_-induced Nox4 mRNA expression was significantly down regulated by AEPS.

### Effects of AEPS on SOD1, CAT and GPx mRNA expression in HUVECs

**Figure 5:**
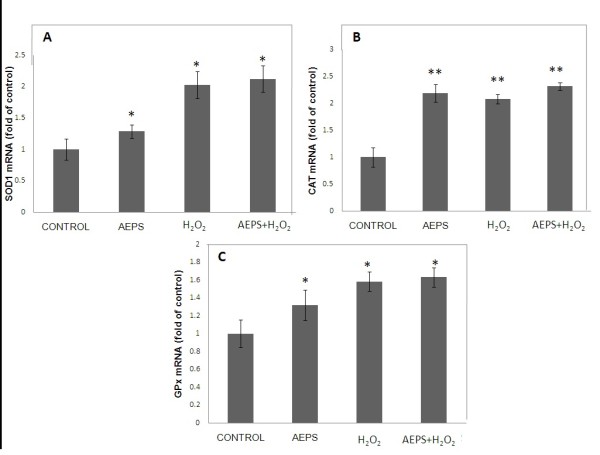
**SOD1, CAT and GPx mRNA expression in HUVECs**. Figure 5 represents the bar chart showing SOD1 (A), CAT (B) and GPx (C) mRNA expression in control, AEPS, H_2_O_2 _and AEPS + H_2_O_2 _groups. Single treatment of HUVEC with AEPS or H_2_O_2 _significantly increased SOD1, CAT and GPx mRNA expression. The highest level of SOD1, CAT and GPx mRNA expression was observed in HUVEC treated with both AEPS and H_2_O_2_. Data are expressed as mean ± SEM of n = 10. (*) p < 0.05 vs. control; (**) p < 0.01 vs. control.

HUVECs treated with AEPS had significantly higher level of SOD1, CAT and GPx mRNA expressions compared to the control group (Figure 5A, 5B, 5C). The highest increase was observed in the CAT expression (2.2-fold) followed by GPx (1.3 fold) and SOD1 (1.2-fold). In the oxidative stress-induced group, HUVECs treated with H_2_O_2 _also showed significantly higher level of SOD1, CAT and GPx mRNA expressions compared with the control group. HUVECs treated with both AEPS and H_2_O_2 _had significantly higher level of SOD1, CAT and GPx mRNA expressions than the control group but no significant difference from the single H_2_O_2_-treated and AEPS-treated groups.

## Discussion

It is well known that the transcription factor NF-κB is essential in regulation of the gene expression of cell adhesion molecules such as VCAM-1, ICAM-1 and E-selectin [5]. Therefore, we hypothesized that AEPS has the ability to modulate the expression of NF-κB and cell adhesion molecules in H_2_O_2_-induced HUVECs. In the present study, both AEPS and H_2_O_2 _did not have any significant effect on the gene expression of NF-κB in HUVECs (Figure 2). In another study, H_2_O_2 _induces NF-κB activation in porcine aortic endothelial cells but not in human aortic endothelial cells, suggesting that porcine endothelial cells might be more sensitive to H_2_O_2 _compared to human endothelial cells [23].

However, treatment with H_2_O_2 _caused an upregulation of ICAM-1 mRNA expression (Figure 3B). The H_2_O_2_-induced ICAM-1 mRNA expression was significantly down regulated by AEPS. Both AEPS and H_2_O_2 _did not have any significant effect on the gene expression of VCAM-1 and E-selectin (Figure 3A, 3C).

In this study, the effects of H_2_O_2 _on the cellular adhesion molecules expression are in accordance with earlier research [24]. In the study, treatment of HUVECs with 50 μmol/L H_2_O_2 _for 24 hours increased the level of ICAM-1 mRNA expression but it did not induce the expression of VCAM-1 and E-selectin. Hydrogen peroxide also did not seem to activate NF-κB. This could be caused by a difference in sensitivity of endothelial cells among different species. Human endothelial cells are relatively resistant to oxidative damage compared to endothelial cells cultured from other species [24]. Treatment with 400 μM H_2_O_2 _upregulates VCAM-1 expression in porcine aortic endothelial cells but not on HUVECs and human aortic endothelial cells, suggesting that porcine endothelial cells might be more sensitive to H_2_O_2 _compared with human endothelial cells [23]. However, at a higher dose (> 1000 μM) of H_2_O_2_, HUVECs can significantly upregulate VCAM-1 expression [23]. The dose used in this study was 180 μM which was much lower than that. The lower dose of H_2_O_2 _and the relative resistance of HUVECs to oxidative damage may contribute to the non-significant changes in VCAM-1 and E-selectin.

The present study demonstrated that AEPS down regulated the mRNA expression of the ROS-producing enzyme Nox4 (Figure 4), and at the same time, upregulated the expression of ROS-inactivating enzymes; SOD1, CAT and GPx (Figure 5A, 5B, 5C) in HUVECs. Although several ROS-generating systems have been described in endothelial and other vascular cells, NADPH oxidases (Nox) have now been recognized to be the major source of ROS in the vasculature [9]. The Nox enzyme complex consists of two essential membrane-bound subunits, gp91phox and p22phox, which composed of cytochrome b558, and several cytosolic regulatory components. The enzyme is dormant in resting cells, but on stimulation, the cytosolic subunits translocate to the cytochrome b558 at the membrane leading to activation of the enzyme and the release of large amounts of superoxides [25].

NADPH oxidase-mediated ROS production is regulated at two levels: gene expression of the Nox subunits and the enzyme activity [26]. To the best of our knowledge, the present study is the first of its kind to report that AEPS decreased the gene expression of Nox4, which is the predominant Nox isoform found in endothelial cells. The present study also showed that treatment of HUVECs with H_2_O_2 _upregulated Nox4 mRNA expression. In another study, H_2_O_2 _was capable of upregulating the Nox subunit p22phox mRNA and protein expression in endothelial cells [25]. The H_2_O_2_-induced Nox4 mRNA expression was significantly down regulated by AEPS (Figure 4). This could be one of the mechanisms by which AEPS reduced endothelial oxidative stress.

The antioxidant enzymes represent a first line of defense against ROS by metabolizing them to innocuous byproducts. The first enzymatic reaction in the reduction pathway of oxygen occurs during the dismutation of two molecules of superoxides when they are converted to H_2_O_2 _and oxygen. The enzyme involved at this step is one of two isoforms of superoxide dismutase (SOD); CuZnSOD or SOD1 which is present in the cytosol while MnSOD or SOD2 is located in the mitochondrial matrix. Although H_2_O_2 _is not a free radical itself, it is reactive and it is rapidly converted into the highly reactive hydroxyl anion in the presence of ferrous ion via the Fenton reaction unless it is efficiently removed. Two enzymes participate in the removal of H_2_O_2 _from the cellular environment; glutathione peroxidase (GPx) and catalase (CAT). Glutathione peroxidase is present in both the cytosol and mitochondria while CAT is present mainly in the peroxisomes. Catalase and GPx detoxify H_2_O_2 _into water and oxygen [12].

Antioxidant enzymes; SOD, CAT and GPx are thought to be effective for augmentation of antioxidant defenses in endothelial cells [27]. As shown in Figure 5, treatment with AEPS upregulated the expression of SOD1, CAT and GPx in HUVECs. These results suggested that the protective effects of AEPS against oxidative stress may be related to the increased ability to upregulate the antioxidant enzymes expression. The greatest inductions were in the levels of CAT mRNA; indicating that CAT plays an important role in scavenging H_2_O_2_. In other reports, upregulation of antioxidant enzymes by resveratrol protects aortic smooth muscle cells [28] and HUVECs [29] against oxidative stress. When HUVECs were exposed to H_2_O_2_, there was an increase in the mRNA expression of SOD1, CAT and GPx. This could be part of the defense mechanism of the cells to protect themselves better from the damage that was induced by H_2_O_2 _[30].

This study showed that AEPS significantly reduced Nox4 expression and increased SOD1, CAT and GPx expression. This indicates an increase in cellular defense mechanism against oxidative stress and implies the vasculature-protective effect of AEPS. Previous study showed that AEPS has direct ROS scavenging ability [31]. Therefore, this study further improves the previous knowledge on the vasculature-protective effects of AEPS as it shows that AEPS can effectively increase cell defense mechanism against oxidative stress.

The leaves of *Piper sarmentosum *contained biologically active flavonoid compounds such as myricetin, apigenin, quercetin and rutin which are potent antioxidants [13,32]. Since the aqueous extract of *Piper sarmentosum *leaves used in the present study was not a purified component, the limitation of this study was the inability to determine the specific components of the plant that mediated the observed effects. However, it is suggested that the effects were due to the flavonoid compounds mentioned above.

## Conclusions

The present study describes some novel effects of AEPS. By decreasing the expression of ICAM-1 and Nox4 and enhancing the expression of SOD1, CAT and GPx, AEPS represents a unique approach in reducing endothelial oxidative stress. The findings indicate a new insight into the mechanism involved in the vascular protective effect of AEPS.

## Competing interests

The authors declare that they have no competing interests.

## Authors' contributions

AU: Performing the study, analyzing the data and preparing the manuscript. ZZ: Supervising the work, providing the grants for the study, evaluating the data, correcting the manuscript and coordinating the study. CKH: Supervising the work, evaluating the data, correcting the manuscript and coordinating the study. NAMMN: Providing the grants for the study, evaluating the data and correcting the manuscript. All authors read and approved the final manuscript.

## Pre-publication history

The pre-publication history for this paper can be accessed here:

http://www.biomedcentral.com/1472-6882/11/31/prepub
